# Temporal Trends in Stillbirth in Greece: A Longitudinal Population-Based Study

**DOI:** 10.7759/cureus.37154

**Published:** 2023-04-05

**Authors:** Nikolaos Vlachadis, Dionysios Vrachnis, Nikolaos Antonakopoulos, Maria Tigka, Nikolaos Loukas, Christos Parthenis, Dimitra Metallinou, Christina Nanou, Sofoklis Stavros, Nikolaos Vrachnis

**Affiliations:** 1 Department of Midwifery, University of West Attica, Athens, GRC; 2 Department of Clinical Therapeutics, National and Kapodistrian University of Athens, Alexandra Hospital, Athens, GRC; 3 Department of Obstetrics and Gynecology, University of Patras, Rio University Hospital, Patras, GRC; 4 Department of Obstetrics and Gynecology, Tzaneio Hospital, Piraeus, GRC; 5 Department of Maternal Fetal Medicine, Elena Venizelou Hospital, Athens, GRC; 6 Third Department of Obstetrics and Gynecology, National and Kapodistrian University of Athens, Attiko Hospital, Athens, GRC

**Keywords:** antenatal care, greece, fetal mortality, fetal death, stillbirth

## Abstract

Introduction: Stillbirths are a major public health issue and a key population health indicator. The aim of this study was to comprehensively investigate and present time trends in stillbirth in Greece.

Methods: Data on all live births and stillbirths were derived from the Hellenic Statistical Authority, covering a 65-year period from 1957 to 2021 and the annual stillbirth rate (SBR) was calculated, defined as the number of stillbirths per 1,000 live births and stillbirths (total births). Trends in the SBR were assessed using joinpoint regression analysis with calculation of the annual percent change (APC) with a 95% confidence interval (95% CI) and level of statistical significance p<0.05.

Results: The SBR in Greece, after an initial increasing trend (1957-1965: APC=2.6, 95% CI: 0.5 to 4.7, p=0.016), and an all-time high of 15.8 per 1,000 births in 1966, recorded a four decades period of continuous improvement (1965-2003: APC=3.0, 95% CI: -3.2 to -2.8, p<0.001) and reached a historic low in 2008 (3.3 per 1,000 births) (a decrease by 79%). However, the SBR stagnated at an elevated level during the decade 2006-2016 and showed a steeply upward trend during the most recent period 2016-2021 (APC=7.4, 95% CI: 3.0 to 12.1, p=0.001). In 2021, the SBR was 5.3 per 1,000 births, 60% up from 2008. It was estimated that the SBR improvement for the 1967-2021 period resulted in 50,914 stillbirths averted (7.9 per 1,000 births), but the recent increase in the SBR has led to 1,200 additional fetal deaths (1.0 per 1,000 births) during 2009-2021.

Conclusion: After an impressive decline for almost four decades the SBR gradually deteriorated during the economic crisis and finally showed an alarming rising trend after 2015, resulting in an increasing burden of fetal deaths in Greece. Further public health interventions are needed to address preventable risk factors and ensure access to optimized antenatal monitoring.

## Introduction

Stillbirth or fetal death is defined as the loss of the fetus at gestational age beyond the limit of viability, which is generally considered to be 24 weeks of gestation in the developed world and 28 gestational weeks in less developed countries. The fetal viability limit has been significantly reduced due to advances in perinatal medicine, and there is no international agreement on gestational age, so the World Health Organization has recommended a cut-off at 28 weeks to facilitate comparisons between countries, whereas in recent years there has been a tendency for fetal losses in the first half of gestation (<20 weeks) to be considered miscarriages, whereas stillbirths are considered those occurring in the second half (≥20 weeks) with a birth weight of at least 350 gr which is the 50th percentile for weight at 20 weeks of gestation [1-3].

Stillbirths constitute a major global health problem with more than two million babies stillborn every year worldwide with a huge psychological burden and economic cost. Furthermore, fetal deaths constitute a neglected tragedy since although they constitute the majority of perinatal deaths, they have not garnered medical and social recognition to a degree comparable to neonatal, infant, and child mortality [4,5]. Stillbirths are in the majority of cases preventable with optimal antenatal monitoring and care, and management of obstetric emergencies [6-8]. The major risk factors associated with the incidence of stillbirth include maternal obesity, advanced maternal age, multiple gestations, smoking, fetal congenital and karyotypic anomalies, medical morbidities such as diabetes and hypertension, and fetal growth restriction, while in developing countries a large proportion of cases are attributed to infections and poor nutrition [1,9,10]. Even in high-income countries, socioeconomic inequalities play a major role in stillbirth epidemiology with women from more deprived socioeconomic backgrounds facing a higher risk [11,12].

The stillbirth rate (SBR) is considered a key health indicator reflecting the socioeconomic development of a population and the level of provision and access to healthcare services around pregnancy and birth within a population [4]. Our aim was to investigate and provide a comprehensive overview of rates and temporal trends of stillbirths in Greece which are plagued by extremely low birth rates [13], high multiple birth rates [14], and advanced maternal age [15].

## Materials and methods

Official national data regarding live births and stillbirths in Greece, based on the birth certificates registered in the country, were retrieved from the Hellenic Statistical Authority, covering a 65-year period from 1957 to 2021, the first and the most recent years with fully available data, respectively.

For each year of the above period, the SBR was calculated, defined as the number of stillbirths per 1,000 live births and stillbirths (total births). Stillbirths for the years 1957-1979 were defined as those that occurred at 28 weeks of gestation or more, whereas stillbirths for the 1980-2021 period were defined as fetal deaths occurring at 20 gestational weeks or more with a minimum fetal weight of 350 gr, in accordance with the definition suggested by the United States Centers for Disease Control and Prevention [1,2].

Trends in the SBR were assessed using Joinpoint regression software version 4.7.0.0 (Surveillance Research Program, National Cancer Institute, Bethesda, Maryland, United States of America). The annual percentage changes (APCs) were calculated with a 95% confidence interval (95% CI) and level of statistical significance (p < 0.05).

We estimated the total number of excess and averted stillbirths due to the change in the SBR. The number of averted stillbirths due to the decline in the SBR during the 1967-2008 and 1967-2021 periods was estimated as the sum of the differences between the annual number of stillbirths that would have occurred if the SBR of the year 1966 had remained unchanged and the annual observed number of stillbirths. Accordingly, the number of excess stillbirths due to the increase in the SBR during the recent years 2009-2021 was estimated as the sum of the differences between the annual observed number of stillbirths and the annual number of stillbirths that would have occurred if the SBR of the year 2008 had remained unchanged. Furthermore, the decline in the number of stillbirths in 2015 compared with 1966 attributable to the decrease in fetal mortality was estimated by applying the 1966 SBR to the total number of births in the year 2015.

Since this was an analysis of national-aggregate publicly available data, ethics board approval or consent procedures were not needed.

## Results

During 1957-2021, a total of 72,672 stillbirths and 7,910,174 live births were registered in Greece with a total of 7,982,846 births (live births and stillbirths), resulting in an overall SBR of 9.1 per 1,000 births.

**Figure 1:**
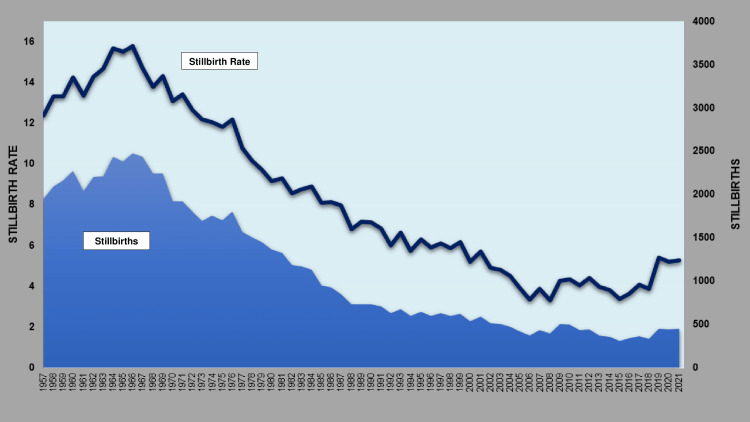
Stillbirths and stillbirth rates (per 1,000 births) in Greece, 1957-2021.

The SBR and the stillbirths for the period 1957-2021 in Greece are depicted in Figure 1.

The SBR increased 27%, from 12.4 per 1,000 births in 1957 to a historic high of 15.8 per 1,000 births in 1966. This was followed by a long period of continuous decline in the SBR, reaching its historic low in 2008 (3.3 per 1,000 births), 79% down from 1966. In the more recent period 2008-2021, the SBR rose again to 5.3 per 1000 births in 2021, an impressive 60% increase.

**Figure 2:**
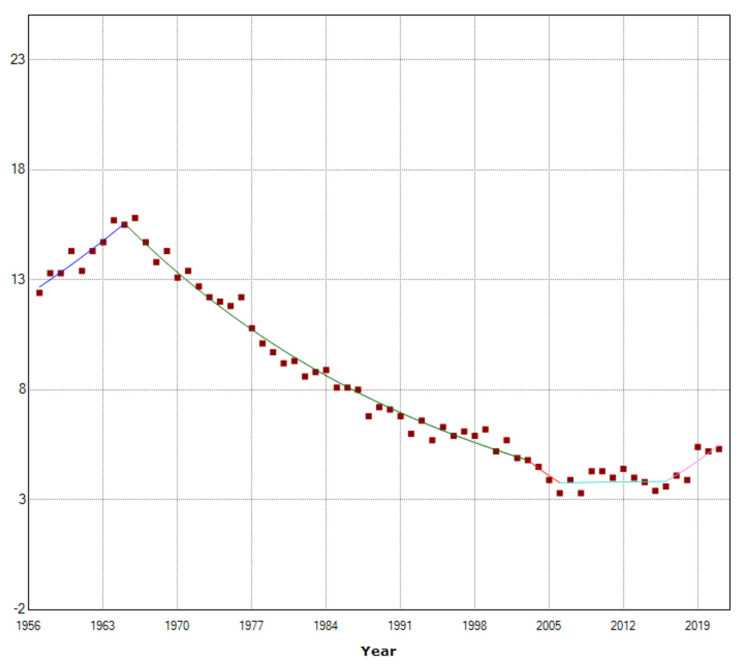
Time trends in stillbirth rates in Greece, 1957-2021.

The time trends of SBR for the 1957-2021 period are presented in Table 1 and Figure 2.

**Table 1:** Time trends in stillbirth rates in Greece, 1957-2021. APC: annual percentage change

Period	APC	95% CI	P-value
1957-1965	2.6	0.5 to 4.7	0.016
1965-2003	-3.0	-3.2 to -2.8	<0.001
2003-2006	-7.7	-23.6 to 11.6	0.402
2006-2016	0.2	-1.6 to 1.9	0.849
2016-2021	7.4	3.0 to 12.1	0.001

The SBR in Greece, after an initial increasing trend (1957-1965: APC=2.6, 95% CI: 0.5 to 4.7, p=0.016) recorded an impressive period of continuous improvement over four decades (1965-2003) with a steady decline of 3.0% per year (95% CI: -3.2 to -2.8, p<0.001). In the three-year period 2003-2006, the decline appeared to accelerate (although non-significantly) (APC=-7.7, 95% CI: -23.6 to 11.6, p=0.402), however, then the improvement of the SBR was halted, and for a decade the SBR stagnated (2006-2016: APC=0.2, 95% CI: -1.6 to 1.9, p=0.849) at an elevated level. Finally, in the last five years 2016-2021, the SBR showed a strong upward trend of 7.4% per year (95% CI: 3.0 to 12.1, p=0.001).

The number of stillbirths increased by 27%, from 1,956 in the year 1957 to an all-time high of 2,479 in 1966, whereas it then fell by 87% to an all-time low of 312 in 2015. If the 1966 SBR had remained unchanged, the expected number of stillbirths for 2015 would have been 1,454. Therefore, of the 2,167 fewer stillbirths, 1,454-312 = 1,142 (53%) are attributed to the decline in SBR, and the remaining 2,167-1,142 = 1,025 (47%) are attributed to the decline in natality in the country. Finally, in the most recent period 2015-2021, stillbirths increased sharply by 45%, reaching 453 in 2021.

**Figure 3:**
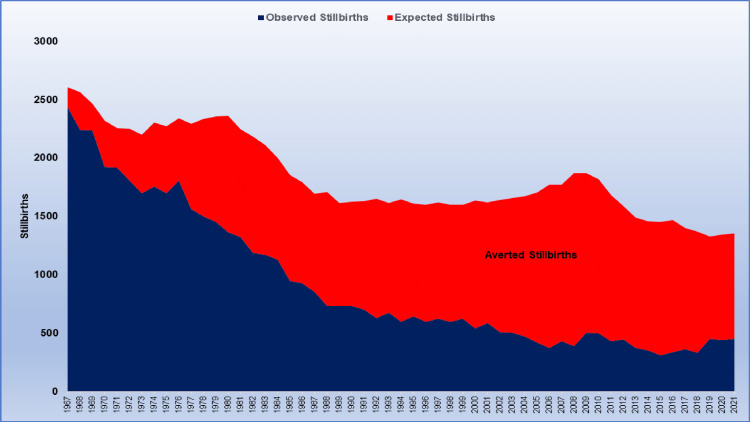
Depiction of the estimated number of stillbirths averted in Greece (red-colored area) due to the improvement in stillbirth rates, 1967-2021.

The reduction in the SBR resulted in 45,110 stillbirths in the period 1967-2008, rather than the 81,693 which would have occurred had the 1966 SBR remained unchanged, meaning 36,583 stillbirths were averted (7.1 per 1,000 births). Similarly, the SBR improvement for the entire period 1967-2021 resulted in 50,420 stillbirths instead of 101,334, thus 50,914 stillbirths were averted (7.9 per 1,000 births) (Figure 3).

**Figure 4:**
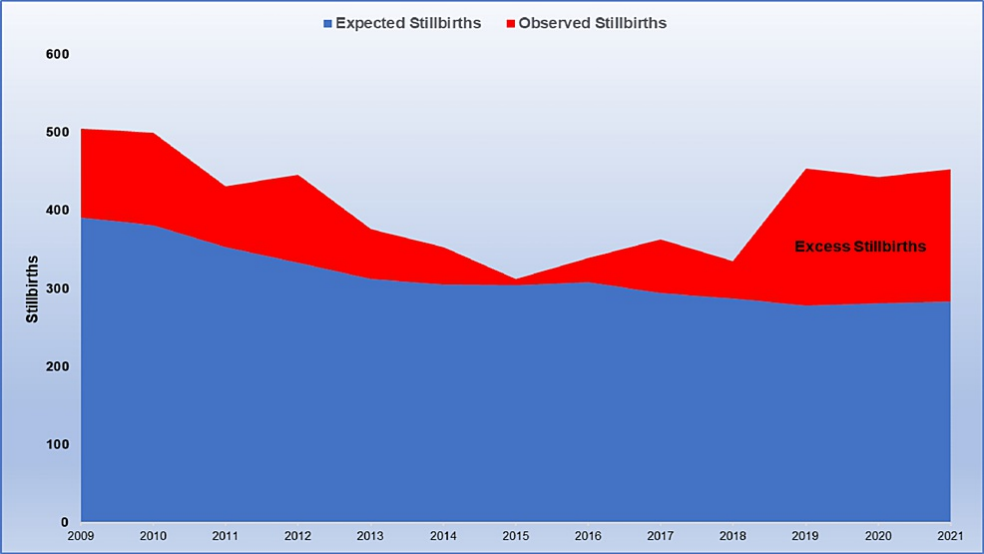
Depiction of the estimated number of excess stillbirths in Greece (red-colored area) due to the deterioration in stillbirth rates, 2009-2021.

In contrast, the increase in the SBR during 2009-2021 resulted in 5,310 stillbirths, instead of the 4,110 we would have occurred if the SBR had remained the same as in 2008, thus there were 1,200 additional stillbirths in the latest 13 years (1.0 per 1,000 births) (Figure 4).

## Discussion

This longitudinal study examined SBR in Greece over more than six decades based on official national population-based data. After an initial rising trend, the SBR in Greece showed a clear downward trend over a long period of four decades, which was halted around the onset of the economic crisis in the country, whereas the SBR has been rising alarmingly since 2015.

In the first decade under investigation, the trend of the SBR was upward with APC=2.6%, reaching an all-time high of 15.8 per 1,000 births in 1966. It is expected that this rising trend largely is attributable to the improving registration of fetal deaths in the country. The problem of under-registration of fetal deaths is international, has been quite severe in previous decades, and seems to have improved considerably in recent years [16].

In 1966, about one in 63 third-trimester pregnancies resulted in fetal loss, however, since 1966, the SBR in Greece has had a steady improvement by 3.0% annually, lasting for nearly four decades. As a result, the SBR in the country reached its all-time low of 3.3 stillbirths per 1,000 total births in 2008, decreasing by 79% or almost five-fold. This improvement was also observed in many other developed countries attributable to advances in prenatal care as well as improving socioeconomic conditions [17,18].

The favorable picture of fetal mortality in Greece was reversed when the country was hit by a huge economic crisis at the end of 2008. The economic recession was associated with the deterioration of all perinatal indicators in the Greek population, but a sharp rise in SBR in 2008-2010 was the first finding in the literature [19]. The present analysis showed that the SBR declined at the historically fastest annual rate of 7.7% in the three-year period 2003-2006 but the trend did not last long enough to become statistically significant, and in the following decade 2006-2016 the SBR stagnated but was elevated. The stabilization of the SBR after the rapid progress of the previous decades is a common finding in many countries with low fetal mortality rates and is in line with a well-known epidemiological phenomenon showing that risk prevention becomes challenging as the incidence reduces [20,21]. However, in Greece, a deterioration of the SBR was observed after 2015 with a large upward trend of 7.4% per year.

The improvement in maternity care has had an impressive impact on public health and perinatal health in the country. The reduction in the SBR resulted in the averting of more than 50,000 fetal deaths during 1967-2021 (around one in 126 babies), and proper maternity care saved about half of the babies who would have died had the SBR remained stable at 1966 levels. Stillbirths declined by 87% from a historic high of 2,479 in 1966 to a historic low of 312 in 2015, an eight-fold decrease. Noteworthy, only about half of this decline is attributable to the decrease in fetal mortality, while the other half is attributable to a decline in fertility in the Greek population. However, the failure to maintain fetal mortality at 2008 levels led to the loss of an additional 1,200 viable babies (approximately one per 1,000) in the more recent period 2009-2021.

Stillbirths in the Greek population have been poorly studied. A study of population data from 1989 to 1991 showed that multifetal pregnancies, low maternal educational level, advanced maternal age, and pregnancies out of wedlock were risk factors for fetal mortality in the Greek population [22]. Other published studies investigated the impact of the recent economic austerity on perinatal parameters, including stillbirths. One study found no statistically significant trends in the SBR in Greece during the pre-crisis (2004-2008) and crisis period (2009-2015), and reported an increased risk for non-Greek mothers which was exaggerated during the crisis compared with the pre-crisis period. Furthermore, statistically significant associations of stillbirth risk with multiple births, birth weight, maternal age, education, and marital status were confirmed [23]. Other researchers also could not identify any significant change in the SBR in Greece during the crisis period but reported an increased risk for mothers <25 years of age compared with the pre-crisis period [24]. Finally, the increased risk of stillbirth among immigrant mothers and those with low education was confirmed by another analysis of national data for the years 2010-2014 [25].

The stagnation of SBR in Greece since the onset of the economic crisis and especially the large increase after 2015 are worrying findings that are related to the deterioration of key risk factors, such as the increase in pregnancies at advanced maternal age, and the increase in twin birth rate [14,15], but also the barriers to access to quality antenatal care services for women of lower socioeconomic level. Especially for the years 2020 and 2021, the possible impact of the COVID-19 pandemic should be taken into account because it has been suggested that the infection of pregnant women increases the risk of intrauterine death, but also because of the exacerbation of the effects of social determinants of health and access to maternal care [26].

This study analyzed the official national data on all births in Greece assessing the burden of stillbirth and quantifying the relative impact of the SBR trends for the first time in the literature. The present analysis is limited by the different gestational age cut-offs used to define stillbirths before and after 1980, and by the under-registration of fetal deaths in Greece. However, the two above-mentioned limitations are highly unlikely to have affected our findings, in fact rather the improvement in SBR over the period 1966-2008 is probably higher. The registration of stillbirths in Greece has improved but it seems that it is still incomplete, especially regarding early fetal deaths (20-27 gestational weeks) which are occasionally recorded as second-trimester miscarriages or not at all, and further efforts in this direction are needed. The SBR in Greece is currently lower than the European average as well as that in the United States [2,23], however, further study is needed on the role of key demographic risk factors in the stillbirth risk in the Greek population, including advanced maternal age and high multiple birth rate. Fetal mortality is the largest component of perinatal mortality, thus it deserves as much attention as that given to reducing neonatal and infant mortality.

## Conclusions

The SBR in Greece decreased drastically for almost four decades reaching a historic low in 2008. However, the SBR remained stagnant during the economic crisis and showed a steeply rising trend after 2015. As the population of women at high risk for stillbirth continues to increase in the country, organized efforts are needed to control risk factors, reduce inequality in perinatal health care related to socioeconomic deprivation, as well as further public health actions to improve access to optimized prenatal monitoring and prompt interventions in high-risk pregnancies.
